# An improved global vegetation health index dataset in detecting vegetation drought

**DOI:** 10.1038/s41597-023-02255-3

**Published:** 2023-05-31

**Authors:** Jingyu Zeng, Tao Zhou, Yanping Qu, Virgílio A. Bento, Junyu Qi, Yixin Xu, Ying Li, Qianfeng Wang

**Affiliations:** 1 Key Laboratory of Environmental Change and Natural Disasters of Ministry of Education, Beijing Normal University, Beijing, 100875 China; 2 College of Environment & Safety Engineering, Fuzhou University, Fuzhou, 350116 China; 3 Research Center on Flood and Drought Disaster Reduction, China Institute of Water Resources and Hydropower Research, Beijing, 100038 China; 4 Universidade de Lisboa, Faculdade de Ciências, Instituto Dom Luiz, 1749-016 Lisboa, Portugal; 5 Earth System Science Interdisciplinary Center, University of Maryland, 5825 University Research Ct, College Park, MD 20740 USA; 6 Zhejiang Institute of Meteorological Sciences, Hangzhou, 310008 China

**Keywords:** Natural hazards, Hydrology

## Abstract

Due to global warming, drought events have become more frequent, which resulted in aggravated crop failures, food shortage, larger and more energetic wildfires, and have seriously affected socio-economic development and agricultural production. In this study, a global long-term (1981–2021), high-resolution (4 km) improved vegetation health index (VHI) dataset integrating climate, vegetation and soil moisture was developed. Based on drought records from the Emergency Event Database, we compared the detection efficiency of the VHI before and after its improvement in the occurrence and scope of observed drought events. The global drought detection efficiency of the improved high-resolution VHI dataset reached values as high as 85%, which is 14% higher than the original VHI dataset. The improved VHI dataset was also more sensitive to mild droughts and more accurate regarding the extent of droughts. This improved dataset can play an important role in long-term drought monitoring but also has the potential to assess the impact of drought on the agricultural, forestry, ecological and environmental sectors.

## Background & Summary

Drought is a complex phenomenon^1^, and can trigger crop failures, food shortages, famines, epidemics, and even mass migration^2,3^. Severe drought events were responsible for widespread negative impacts on natural and socioeconomic systems^4,5^. Therefore, it is urgent to improve our understanding of the spatiotemporal characteristics and the evolutionary trends of droughts^6,7^. This would provide a basis for quantifying drought impacts and the social, economic and natural ecological responses to droughts across regions and times^8^.

According to different subjects, drought usually presents four processes or types: meteorological (insufficient precipitation), agricultural, also called vegetation drought (insufficient soil moisture), hydrological (insufficient runoff and/or groundwater), and socioeconomic (social response to water supply and demand)^9^. Among these, vegetation drought is highly correlated with food production, and the extent of vegetation drought have been increasing^10,11^.The global average harvested area has decreased by 4.1% during droughts from 1964–2007^12^. The number and duration of severe drought events in the world are expected to maintain its growth in the future^13,14^. An efficient technique to detect vegetation drought events and monitor the potential risk of droughts in the long term is thus urgently needed^15,16^. This may be seen as a key asset to help policymakers implement timely mitigation and adaptation strategies^17^.

Traditional meteorological drought index has been widely used for vegetation drought assessment^18–20^. For example, the self-calibrating Palmer drought severity index (sc-PDSI)^21^ significantly improves spatial homogeneity, taking into account soil moisture conditions. Four values related to the soil moisture are computed along with their complementary potential values. These eight values are evapotranspiration (ET), recharge (R), runoff (RO), loss (L), potential evapotranspiration (PE), potential recharge (PR), potential runoff (PRO), and potential loss (PL). The PDSI depends on a two-stage “bucket” model of the soil^22^. The top layer of soil is assumed to hold one inch of moisture. The amount of moisture that can be held by the rest of the underlying soil is a location-dependent value, which must be provided as an input parameter to the program. The sc-PDSI automatically calibrates the behavior of the index at any location by replacing empirical constants in the index computation with dynamically calculated values^21^. Compared with other meteorological drought indices [such as the standard precipitation index (SPI) and the standardized precipitation-evapotranspiration index (SPEI)], the sc-PDSI has a higher correlation with vegetation drought^23^. However, these indices still face problems such as inaccurate station observation and delayed data collection^7,24^, and do not consider either the impact of water and heat stress on vegetation growth or do not fully consider land cover and vegetation information of the underlying surface^25^. Therefore, the traditional meteorological drought index is not suitable for direct use in vegetation drought research. But using it as an auxiliary information supplement tool and combining it with other relevant technologies can help us better understand and study vegetation drought. Currently, satellite data are widely used for drought assessment, because of its ability to identify drought conditions on different underlying surfaces^26^. Remotely sensed drought monitoring has advantages such as being suitable for large-scale drought monitoring^27^, having a powerful real-time update function^28,29^, and high accuracy, as well as unmatched cost-effectiveness when compared to other methods^30,31^. Therefore, the combination of satellite remote sensing technology and traditional drought index is a very promising drought assessment method.

The vegetation health index (VHI) is one of the most popular remote sensing drought monitoring indices^32–35^. VHI is composed by two terms: the vegetation condition index (VCI) and the thermal condition index (TCI). VHI considers local biophysical and climatic conditions, and can be used for actual plant drought monitoring in various agrometeorological regions^36^. The basic principles of VHI are as follows: (1) a low normalized differences vegetation index (NDVI) and high land surface temperature (LST) suggest poor vegetation health^37,38^; and (2) the contributions of VCI and TCI to VHI are assumed to be equal, since there are no data on the relative contributions of other conditions to vegetation health^35^. However, the contributions of TCI and VCI to VHI depend on climatic and other environmental factor^39^. Indeed, environmental conditions in different regions are usually different, which implies that giving equal weights to VCI and TCI may reduce the application prospects of the VHI, increasing the uncertainty in drought detection^33^. Therefore, evaluating the contributions of VCI and TCI to VHI while considering the underlying surface and hydrothermal conditions is a key issue that needs to be addressed. Bento, *et al*.^39^ used SPEI index combined with Pearson correlation analysis to evaluate the contribution of TCI and VCI to VHI in arid regions, while Zeng, *et al*.^23^ compared SPEI and sc-PDSI as a control drought index to evaluate the contribution of TCI and VCI to VHI.

The purpose of this study is to produce a global, long-term (1981–2021), high-spatial-resolution, improved vegetation drought detection dataset and a reference parameter dataset for calculating the improved VHI for different regions. Here, we supplemented meteorological and soil moisture information on the basis of the VHI of the original algorithm and used the sc-PDSI as the control drought index, combined with the Pearson correlation analysis method, to evaluate the best contributions of VCI and TCI to VHI in different regions. Then, we generated a global long-term dataset for detecting vegetation drought. We also used drought event records from the Emergency Events Database (EM-DAT) to assess the drought detection ability of our improved VHI dataset. The developed global vegetation drought dataset has the potential to monitor and assess drought and its impact on the agricultural, forestry, ecological, and environmental sectors.

## Data and Methods

### Dataset coverage

**Fig. 1:**
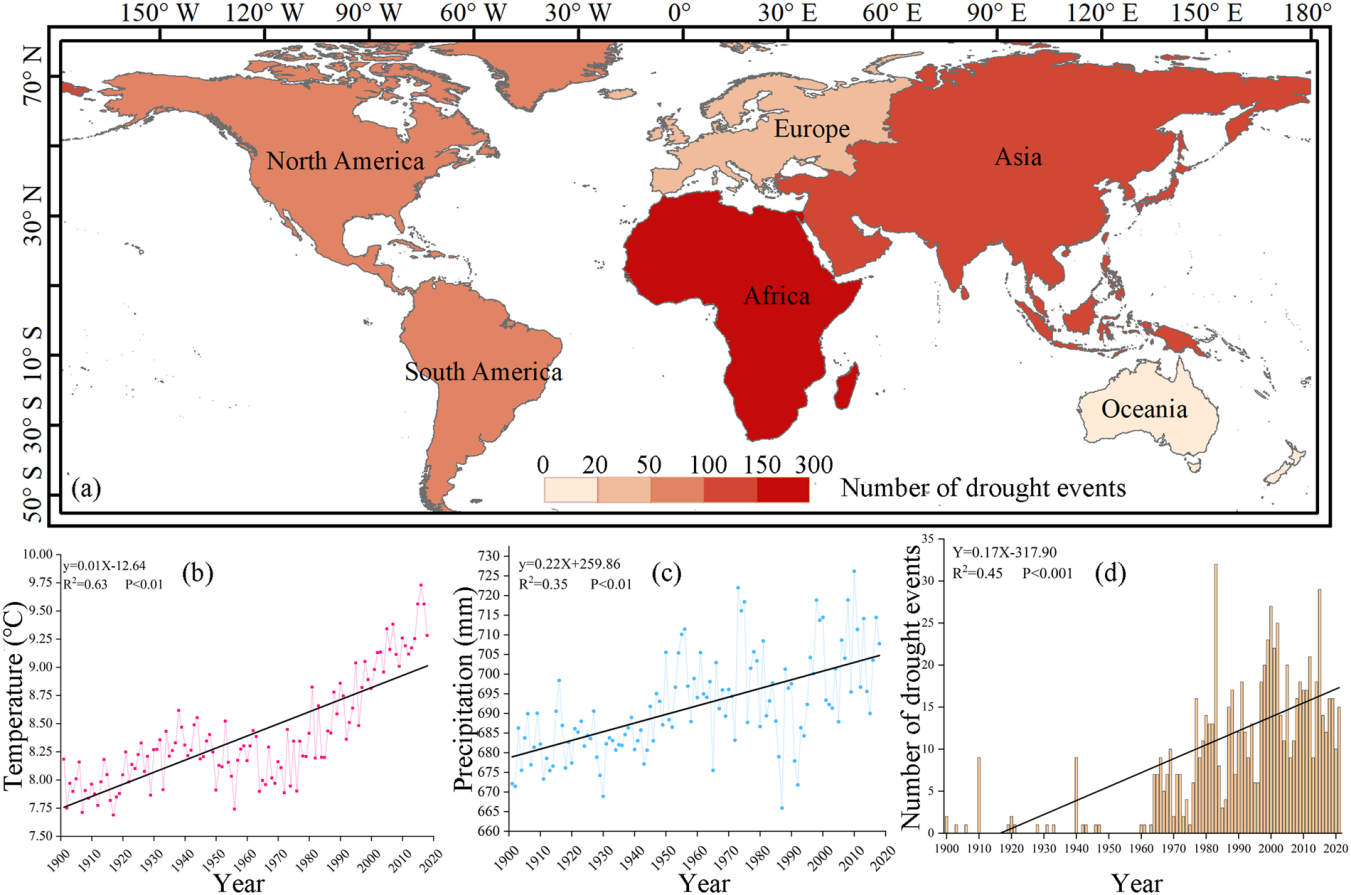
Dataset Coverage (**a**) Number of global drought events, (**b**) the change in global annual mean temperature, (**c**) the change in global annual precipitation, and (**d**) the change in the number of global annual droughts. The black lines in (**b**–**d**) are the best-fit lines from linear regression. The relations, coefficients of determination, and p-values are given in each panel. The data in a and d are sourced from EM-DAT, and the data in b and c are sourced from CRU.

Dataset coverage ranges from approximately from −50°S to 70°N and from −180°W to 180°E. Antarctica, the high latitude regions of the Northern Hemisphere (due to the lack of VCI and TCI data in this area), and the Sahara Desert region in Africa (due to the lack of sc-PDSI in this area) were excluded from the study area. These areas have little vegetation or extremely low vegetation coverage, so this part is usually excluded when studying vegetation drought^40–43^, and these areas are not considered in this dataset. The colors on Figure 1 represent the number of drought events since 1900. Figure 1 also shows information on the change in global annual mean temperature (Fig. 1b), change in global annual precipitation (Fig. 1c), and the change in global annual number of droughts (Fig. 1d). Since the 20th century, global temperatures have substantially increased, and precipitation also showed a positive trend, but with high fluctuations and large uncertainties. Significantly higher temperatures and greater uncertainty in precipitation could dramatically increase drought risk. According to statistics, drought events have been on the rise since 1900 (*p* < 0.001), and especially since the 1980s.

### Data sources

Global VCI and TCI data were downloaded from the National Oceanic and Atmospheric Administration (NOAA) Center for Satellite Application and Research (STAR) (https://www.star.nesdis.noaa.gov/smcd/emb/vci/VH/vh_ftp.php), with a spatial resolution of 4 km, a weekly temporal resolution, and a time span ranging from 1981 to 2021. We organized the data in annual values through the arithmetic mean, and processed the background value (−9999) as a null value through the spatial masking technique to facilitate subsequent spatial and related statistical analyses.

Global sc-PDSI data were downloaded from the Climate Research Unit (CRU), with a spatial resolution of 0.5°, a monthly temporal resolution, and a time span ranging from 1901 to 2020 (https://crudata.uea.ac.uk/cru/data//drought/#global)^44,45^. We organized the data in annual data by the arithmetic mean, then resampled to 4 km by the nearest neighbor method to match the VCI and TCI data. Resampling the 0.5 ° sc-PDSI to 4-km will not lose the information it contains, making the results acceptable. However, the use of sc-PDSI with higher spatial resolution may provide more information on hydrothermal and soil characteristics and may reduce the uncertainty of results.

Data on global drought events from 1900 to 2021 were obtained from the EM-DAT (https://public.emdat.be/)^46^. After filtering and eliminating invalid data, a total of 592 valid records from 1981 to 2021 were selected. We have uploaded the drought event data records for verification to figshare, which can be obtained from 10.6084/m9.figshare.19811854.v5^47^.

### Annual VHI calculation

According to Kogan^35^, the VCI for each pixel and period in a given year was calculated as follows:

1 $$VCI=\frac{NDVI-{NDVI}_{MIN}}{{NDVI}_{MAX}-{NDVI}_{MIN}}\times 100$$

where NDVI is the value of a given pixel and period, and *NDVI*_*MIN*_ and *NDVI*_*MAX*_ are the minimum and maximum values of NDVI for all pixels and periods, respectively. Equation (2) was used to calculate the TCI:

2 $$TCI=\frac{{LST}_{MAX}-LST}{{LST}_{MAX}-{LST}_{MIN}}\times 100$$

where LST is the value of a given pixel and period. *LST*_*MIN*_ and *LST*_*MAX*_ are the minimum and maximum values of LST for all pixels and periods, respectively. The VHI represents the overall health of the vegetation and is used to identify drought^48^. It is calculated by combining the VCI and the TCI as follows:

3 $$VHI=a\times VCI+(1-a)\times TCI$$

where *a* determines the contributions of VCI and TCI to VHI, which varies depending on the environment of the study area^33^. The original VHI (VHI_ori_) assumes equal contributions from water demand (here, a proxy of NDVI) and temperature during plant growth, and the coefficient *a* is assigned the value of 0.5. Following other studies^35,49,50^, we classified drought levels on the basis of the VHI (Table 1):

**Table 1:** Vegetation drought classification based on the VHI.

Category	VHI
Extremely dry	[0, 10]
Severely dry	(10, 20]
Moderately dry	(20, 30]
Mild dry	(30, 40]
Normal	(40, 50]
Good	(50, 60]
Excellent	(60, 100]

### Improvement of the VHI algorithm

We chose the meteorological drought index sc-PDSI, which considers hydrothermal conditions and soil moisture, as the control drought index, and combined it with Pearson correlation analysis to evaluate the contribution of VCI and TCI on a grid by grid basis, thereby obtaining an improved VHI index. To improve VHI, the following steps are taken: (1) The parameter *a* was set to vary in steps of 0.02, starting from 0.02 and gradually increasing to 0.98 (49 values in total). The VHI was then calculated in each step for each year from 1981 to 2021 based on different *a* values. The specific formula is as follows:

4 $${VHI}_{i,t,a}=a\times {VCI}_{i,t}+(1-a)\times {TCI}_{i,t}(a=0.02,0.04,\ldots 0.98)$$

Where *VHI*_*i,t,a*_ represents the VHI when the VCI contribution of the ith pixel is *a* at time t. *VCI*_*i,t*_ represents the VCI of pixel i at time t, and *TCI*_*i,t*_ represents the TCI of pixel i at time t.

(2) The Pearson correlation was then used to evaluate the correlation between sc-PDSI and the VHI calculated in each iterative step of contribution values *a* pixel by pixel. The sc-PDSI based on the water balance theory fully considers meteorological factors and soil moisture conditions, suitable for improving the VHI^23^. The Pearson correlation coefficient is calculated as follows:

5 $${R}_{i}=\frac{{\sum }_{i=1}^{n}\left({x}_{i}-\overline{{x}_{i}}\right)\left({y}_{i}-\overline{{y}_{i}}\right)}{\sqrt{{\sum }_{i=1}^{n}{\left({x}_{i}-\overline{{x}_{i}}\right)}^{2}}\sqrt{{\sum }_{i=1}^{n}{\left({y}_{i}-\overline{{y}_{i}}\right)}^{2}}}$$

Where *R*_***i***_ represents the correlation coefficient between VHI and sc-PDSI of pixel i, *x*_*i*_ represents the VHI of pixel i, $\overline{{x}_{i}}$ represents the average value of VHI of pixel i from 1981 to 2021, *y*_*i*_ represents the sc-PDSI of pixel i, and $\overline{{y}_{i}}$ denotes the mean value of sc-PDSI for pixel i from 1981 to 2021. The range of |*R*| is 0 to 1.

(3) After obtaining the spatial correlation coefficient map of sc-PDSI and VHI calculated with different *a* values, the optimal contribution value *a* was estimated pixel by pixel according to the following formula:

6 $${a}_{i,opt}=MAX\;R({VHI}_{i,a},{scPDSI}_{i})$$

Where *a*_*i,opt*_ represents the best contribution value *a* of VCI to VHI at pixel i, and (1-*a*_i,opt_) represents the best contribution value of TCI to VHI. R is the correlation coefficient between VHI and sc-PDSI, *VHI*_*i,a*_ is the VHI of pixel i when the contribution value of VCI is *a*, and *scPDSI*_*i*_ is the sc-PDSI of the ith pixel. By comparing the correlation coefficients between sc-PDSI and VHI calculated for the 49 values of *a* pixel by pixel, the *a* value of the largest correlation coefficient was selected as the best contribution value *a* of the VCI to VHI of this pixel.

(4) Finally, according to the best contribution value a and the calculation formula of VHI, the improved VHI, namely VHI_opt_, is obtained. The specific formula is as follows:

7 $${VHI}_{i,t,opt}={a}_{i,opt}\times {VCI}_{i,t}+(1-{a}_{i,opt})\times {TCI}_{i,t}$$

**Fig. 2:**
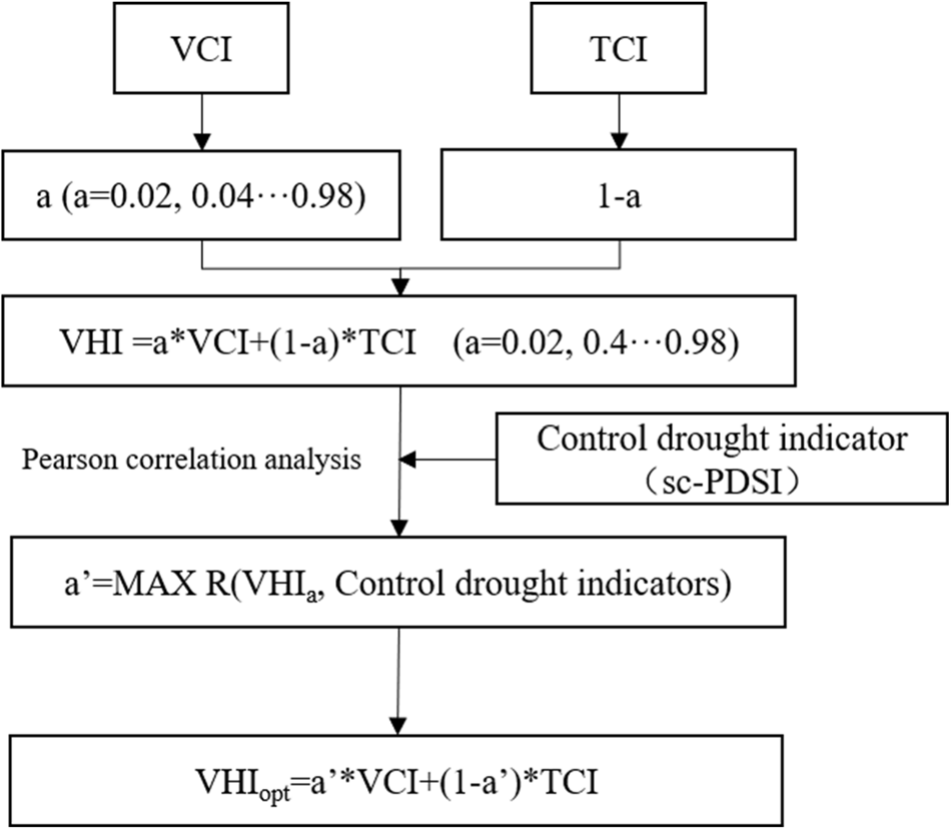
Flowchart of improving VHI index based on sc-PDSI and Pearson correlation analysis.

Where *VHI*_*i,t,opt*_ represents the VHI obtained by the best contribution value *a* of the ith pixel at time t, and *a*_*i,opt*_ represents the best contribution value of the VCI to the VHI of the ith pixel. The algorithms used to improve the VHI index have been uploaded to Github and can be obtained from https://github.com/BNUJingyuZeng/A-new-global-VHI-dataset-code. A general overview of the working scheme is given in Fig. 2.

We compared the scatterplots and linear regression fittings of sc-PDSI and VHI before and after the improvement using detrending. The method of detrending is as follows:

8 $${VHIde}_{i,t}={VHI}_{i,t}-{VHI}_{i,t-1}$$

Where ***VHIde***_***i****,****t***_ represents the VHI for the ith pixel at time t after detrending, *VHI*_*i,t*_ represents the VHI for ith pixel at time t before detrending, and *VHI*_*i,t*−1_ represents the VHI of pixel ith at time t-1 before detrending.

### Evaluation of the drought detection efficiency

Drought detection efficiency of the VHI dataset was evaluated before and after the improvement, based on drought event records in the EM-DAT. The specific formula is as follows:

9 $$DTE=\frac{s}{TDE}\times 100$$

DTE is the drought detection efficiency, S is the score, and TDE is the total number of drought events. The score is evaluated according to the following principles. The VHI-based vegetation drought rating scale threshold is selected as 40. When the VHI is lower than 40, this means dryness (Table 1) - let’s assume drought, and if it is higher than 40, this means normal or wet conditions (Table 1). Given the occurrence time and location of the drought event, VHIs before and after the improvement are compared one by one. When the number of pixels detected by drought accounts for more than 80% of the number of pixels in the area where the drought event occurred, the score is 1, when the number of pixels detected by drought accounts for more than 40% of the number of pixels in the area where the drought event occurred, the score is 0.5, when the number of pixels detected by drought accounts for less than 40% of the number of pixels in the area where the drought event occurred, the score is 0.

### Statistical methods

The Mann–Kendell (MK) method is widely used in meteorology, ecology, environmental research^51–54^. It is a nonparametric test method^55,56^. We used the Theil-sen trend analysis and the MK trend detection methods to study the temporal and spatial trends of global vegetation drought.

## Data Records

### Extent, projection, resolution and data format

The VHI_opt_ dataset covers most of the world’s land areas except Antarctica, with an approximate range of −50°S to 70°N and −180°W to 180°E. The data projection is GCS_WGS_1984. This dataset is available as a 4-km GeoTIFF accessible from a data repository on figshare (10.6084/m9.figshare.19811854.v5)^47^. For each year’s VHI_opt_ image, the globe is divided into 10000 × 3616 1-km × 1-km grid cells. In addition to annual data, we also provide monthly data, which can be used to analyze vegetation drought and related research from the seasonal scale analysis, and also provide a list to explain which months’ data are missing due to the lack of data, and how to combine weekly data into monthly data. We also provide a global best contribution parameter dataset based on the 1981–2021 VHI dataset, i.e., the *a*_*opt*_ parameter file. The file’s extent, projection, spatial resolution and data format are consistent with the VHI_opt_ dataset. The annual VHI_opt_  dataset does not exceed 12 GB (about 3 GB for the compressed download). To preserve as much information as possible, we kept the value of each cell to six decimal places. Missing data are represented by “*Nodata*”.

### Data naming and availability

The global 1981–2021 VHI_opt_ dataset is named “VHIopt_year.tif”, where year corresponds to the year of the data, with a total of 41 files. The VHI_opt_ data of the corresponding year is calculated from annual VCI and TCI data of the year with the best contribution parameter graph from the *a*_*opt*_ file. The parameter file of the global best contribution value a is directly named *a*_*opt*_, which can be used to calculate VHI data from small to macro scales and from daily to seasonal scales.

## Technical Validation

### Drought detection efficiency of the VHI_opt_ dataset

**Fig. 3:**
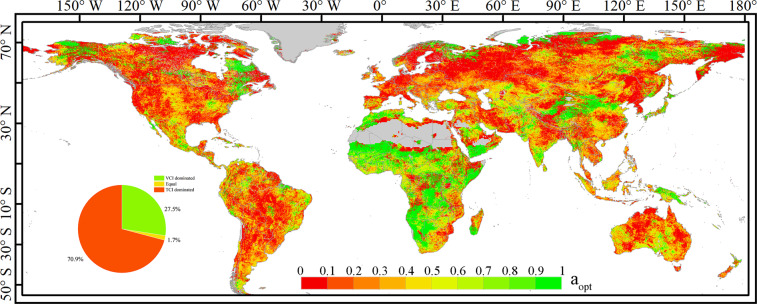
Global distribution of the best contribution rate ***a***_***opt***_ of global VCI to VHI_opt._ The pie chart shows the proportion of regions dominated by TCI (red) or VCI (green).

The best contribution value *a*_*opt*_ is less than 0.5 in most regions of the world. The proportion of regions dominated by TCI reaching 70% and the proportion of regions dominated by VCI of ~28% (Fig. 3). VHI_opt_ is affected by TCI and VCI differently in different regions. Most of Africa is dominated by VCI, while South America, Australia, and regions north of 30° latitude are dominated by TCI. This is especially visible in Europe, where TCI-dominated regions are concentrated and contiguous, with *a*_*opt*_ values generally lower than 0.3. Overall, an abnormally high surface temperature is the main driving factor affecting vegetation drought.

**Fig. 4:**
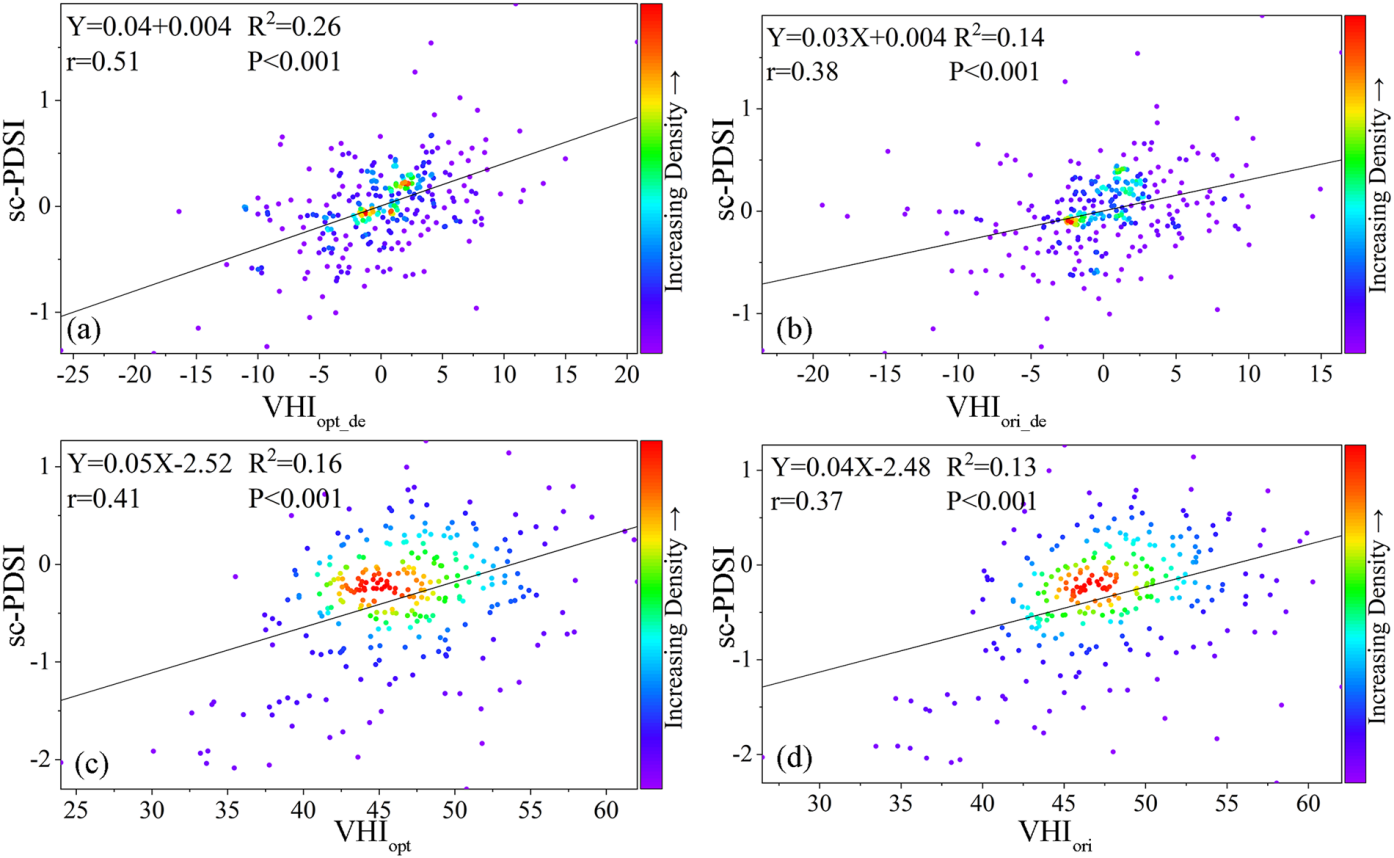
Scatter plots of sc-PDSI as a function of (**a**) detrended VHI_opt_, (**b**) detrended VHI_ori_, (**c**) VHI_opt_, and (**d**) VHI_ori_. The black lines are the best-fit lines from linear regression. The relations, correlation coefficients, and p-values are given in each panel.

The coefficient of determination of the VHI before and after the improvement and the sc-PDSI were compared, as well as the performance before and after detrending (Fig. 4). The coefficient of determination of VHI_opt_ and sc-PDSI (Fig. 4a,c) agree better compared with VHI_ori_ (Fig. 4b,d). The correlation between VHI_opt_ and sc-PDSI was 0.13 higher than that between VHI_ori_ and sc-PDSI (i.e., 0.51 and 0.38, respectively) after excluding time-dependent trends (Fig. 4a,b), which implies an improvement of 34% in correlation. The correlation between VHI_opt_ and sc-PDSI without detrending was 0.04 higher than that between VHI_ori_ and sc-PDSI (i.e., 0.41 and 0.37, respectively; Fig. 4c,d), i.e., an improvement of 10%. The improved VHI dataset can better capture soil moisture anomalies and heat stress levels than the original VHI dataset, thus improving the detection of vegetation drought.

**Fig. 5:**
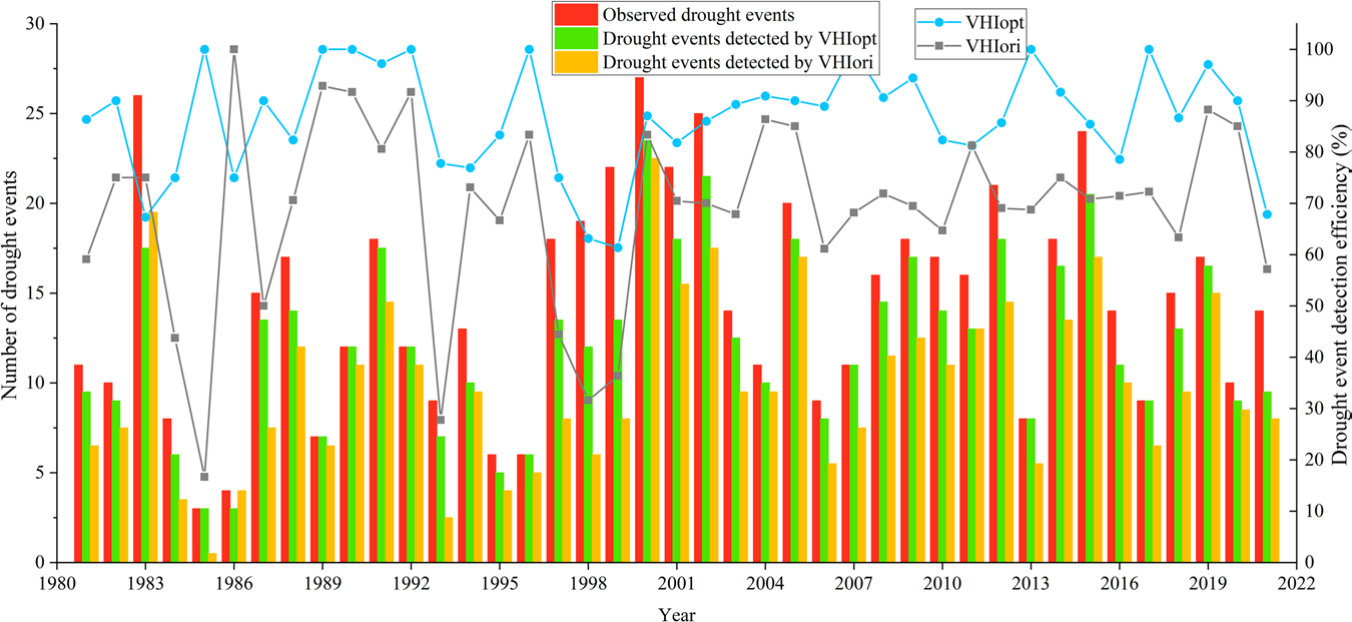
Histogram of the number of observed (red), VHI_opt_-detected (green), and VHI_ori_-detected (orange) global drought events from 1981 to 2021. The blue and dark gray lines show the drought detection efficiencies (unit: %) of VHI_opt_ and VHI_ori_, respectively.

Based on global drought events recorded in the EM-DAT, we compared the ability of VHI_opt_ and VHI_ori_ to detect drought in a year-by-year globally (Fig. 5). The ability of VHI_opt_ to detect drought was higher than that of VHI_ori_, with drought detection efficiencies about 84.97% and 70.69%, respectively. The period from 1997 to 2002 was a period of frequent drought events in the world. The year 2001 experienced the largest number of drought events, i.e., 27. Drought events have declined in recent years.

**Fig. 6:**
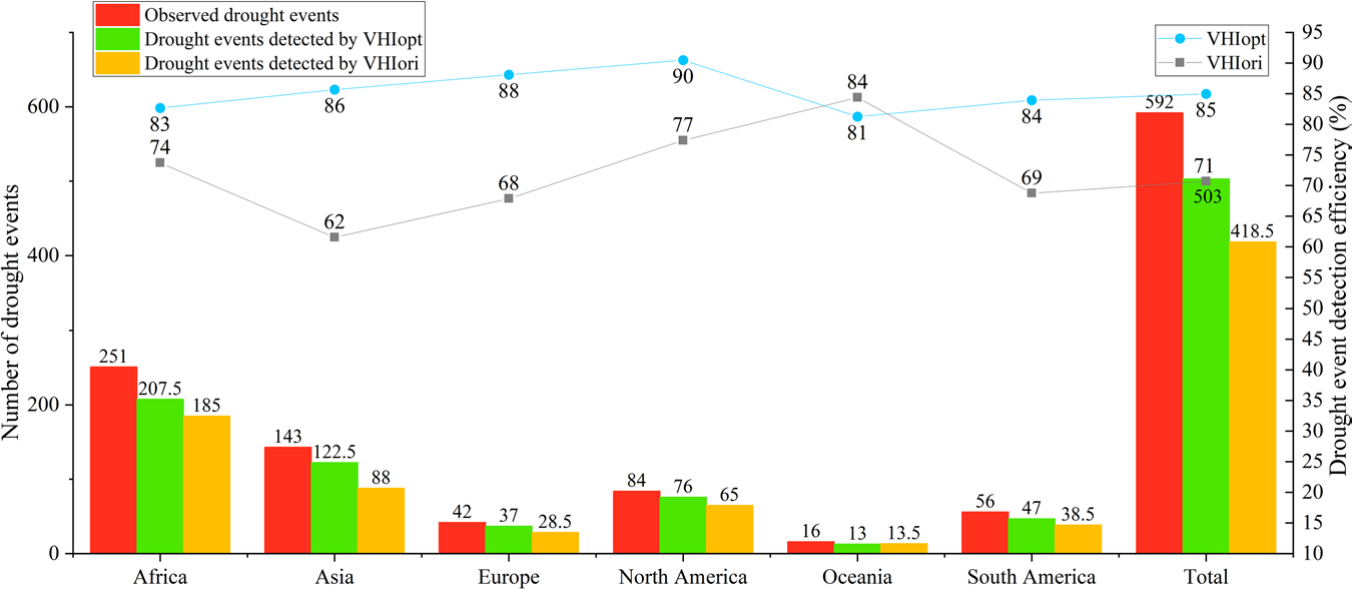
Histogram of the number of observed (red), VHI_opt_-detected (green), and VHI_ori_-detected (orange) drought events from 1981 to 2021 in different continents. The blue and gray lines show the drought detection efficiencies (unit: %) of VHI_opt_ and VHI_ori_, respectively.

The same analysis was repeated but by continent (Fig. 6). The drought detection efficiency of VHI_opt_ was higher than that of VHI_ori_ in all continents except Oceania. The drought event frequency from high to low was 251, 143, 84, 56, 42, and 16 for Africa, Asia, North America, South America, Europe, and Oceania, respectively. The VHI_opt_ drought detection efficiency was highest in North America at 90% and lowest in Oceania at 81%. The VHI_ori_ drought detection efficiency is the highest in Oceania at 84% and lowest in Asia at 62%. The drought detection efficiencies of VHI_opt_ and VHI_ori_ in Oceania were not much different, related to the small number of drought events occurring there. In addition, in many areas of Oceania, drought occurs on small islands, which are affected by the resolution of the data. In Australia, the drought detection efficiency of VHI_opt_ was still higher than that of VHI_ori_.

In general, compared with the original VHI_ori_, our improved VHI_opt_ dataset has an improved drought detection efficiency in all continents. It has broad application prospects for the detection and long-term monitoring of vegetation drought.

### Spatial differences between VHI_opt_ and VHI_ori_

**Fig. 7:**
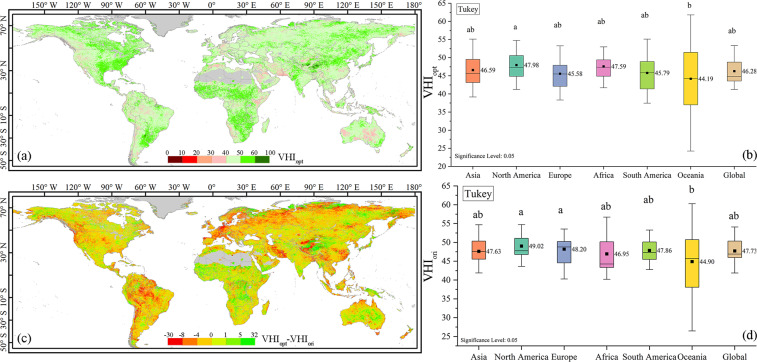
Global distributions of (**a**) VHI_opt_ and (**c**) differences between VHI_opt_ and VHI_ori_. Box plots of (**b**) VHI_opt_ and (**d**) VHI_ori_ globally and by continent. The letters above the boxes indicate significant differences at the p = 0.05 level.

Based on the estimated global best contribution value *a*_*opt*_, we analyzed global annual VHI_opt_ and VHI_ori_ from 1981 to 2021 and compared their spatial patterns (Fig. 7). A relatively low degree of global vegetation drought is seen, with normal to good levels in most areas over the past 40 years, especially near the Equator, eastern North America, and southeastern South America. Clear differences between the two indices are seen (Fig. 7c). VHI_opt_ effectively detected mild vegetation drought, while VHI_ori_ underestimated the occurrence and impact range of mild vegetative drought. VHI_ori_ was significantly higher than VHI_opt_ along the western coasts of North America, Europe, South America, and southwest Asia.

We also compared VHI_opt_ and VHI_ori_ globally and by continents (Fig. 7b,d). VHI_ori_ is higher than VHI_opt_ in 66.5% of the cases. Oceania was the region with the lowest level of vegetation health in the world, as well as the region with the greatest variability, the highest uncertainty, and a higher risk of vegetation health. The vegetation health level in North America was the highest in the world, with average values of 49.02 and 47.98 for VHI_ori_ and VHI_opt_, respectively, which are significantly different from Oceania (*p* < 0.05).

**Fig. 8:**
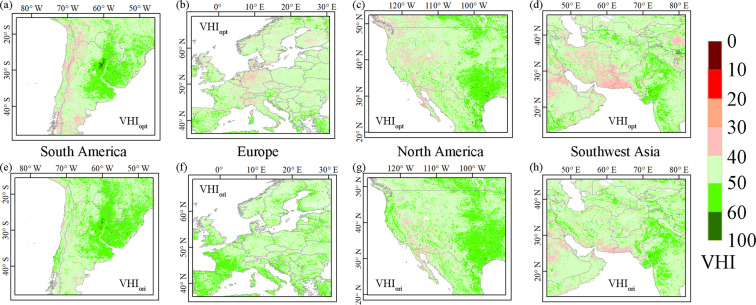
Spatial distributions of (**a**–**d**) VHI_opt_ and (**e**–**h**) VHI_ori_ in South America, Europe, the American Midwest, and southwest Asia, respectively.

We further zoomed in on the above regions to compare spatial differences between VHI_opt_ and VHI_ori_ (Fig. 8). In South America and Europe, VHI_opt_ values below 40 indicates that this index effectively detected vegetation drought, especially in specific years. VHI_ori_ values above 40 indicate normal conditions. VHI_opt_ was more sensitive to mild vegetation drought than VHI_ori_, illustrating its better ability to detect mild drought. VHI_opt_ also improved the ability to assess the occurrence range of vegetation drought compared with VHI_ori_. For example, in the western United States and southwestern Asia, the spatial range of VHI_ori_ values below 40 was smaller than that of VHI_opt_, and there was an overly optimistic estimate of vegetation health. Furthermore, VHI_opt_ also more effectively assessed the extent of vegetation drought in specific years. In general, VHI_opt_ has clear advantages over VHI_ori_ in both drought detection efficiency and drought occurrence range identification.

### Trends seen in the global VHI_opt_ dataset

**Fig. 9:**
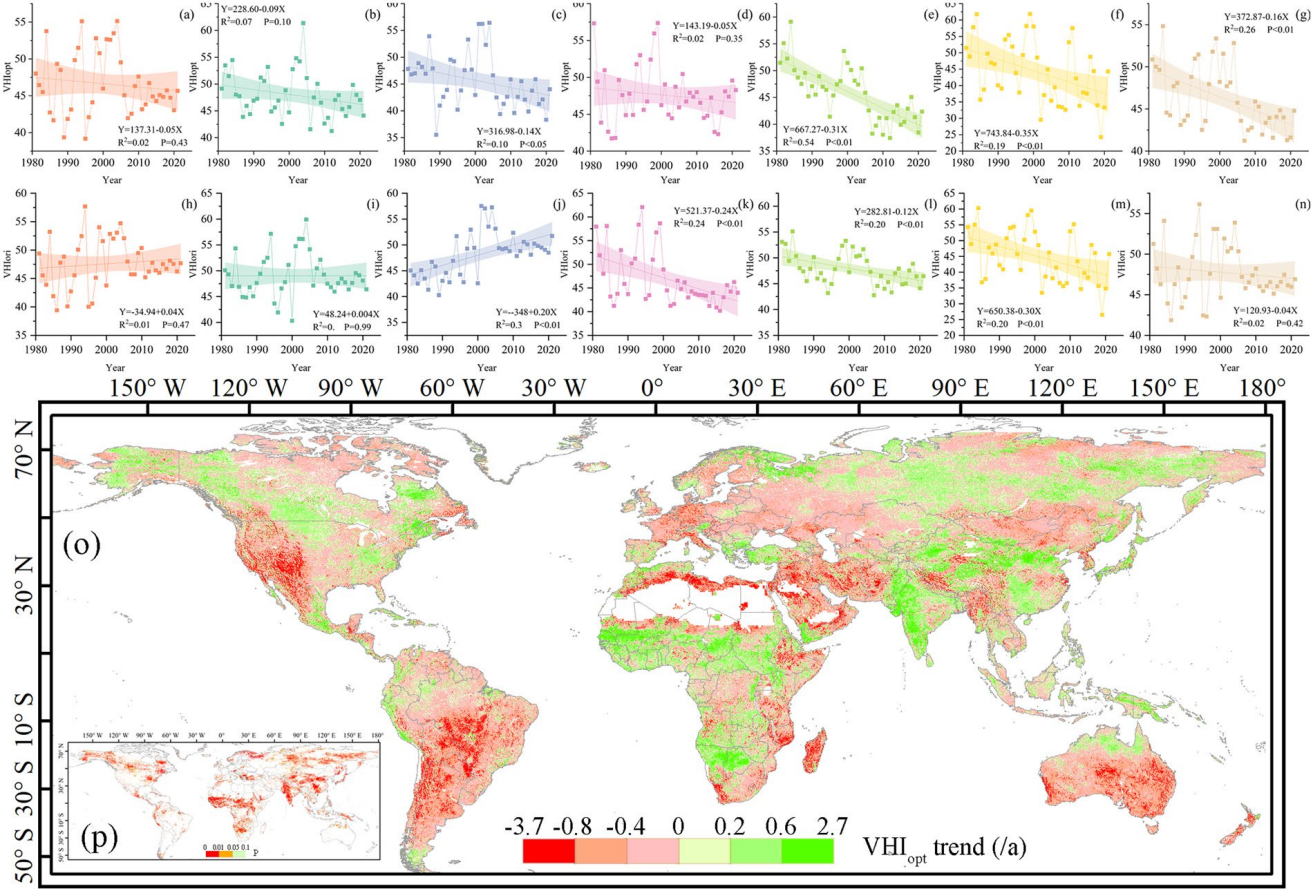
Time series of (**a**–**g**) VHI_opt_ and (**h**–**n**) VHI_ori_ in Asia, North America, Europe, Africa, South America, Oceania, and globally, respectively. Global (**o**) trends in VHI_opt_ (unit: per year) and (**p**) p-values. The relations, coefficients of determination, and p-values are given in each (**a**–**n**) panel.

We analyzed global and continental trends in VHI_opt_ and VHI_ori_ from 1981 to 2021 (Fig. 9). Results show that over the past 40 years, the VHI_opt_ in Europe, South America, and Oceania has decreased significantly by about 0.14, 0.31, and 0.35 per year, respectively. There was no significant change in Asia, North America, and Africa. Globally, VHI_opt_ showed an overall downward trend, with an average annual decrease of 0.16. VHI_ori_ showed a significant increase in Europe, with an annual increase of about 0.2, and a significant decline in Africa, with a decrease of about 0.24 per year. A further analysis showed that South America was the region with the most obvious downward trend in VHI_opt_ (R^2^ = 0.54). From 1981 to 2005, inter-annual differences in Asia were relatively large, and from 2005 to 2021, changes in VHI_opt_ and VHI_ori_ gradually stabilized. This suggests that the future uncertainty of vegetation health in Asia may be less than in other regions.

Since VHI_opt_ has noticeable advantages over VHI_ori_ in both drought detection efficiency and drought occurrence range identification, we used the VHI_opt_ dataset to evaluate the changing trend of global vegetation drought to identify hot spots where vegetation drought may be further aggravated in the future and areas with improved vegetation health (Fig. 9). Results show that the spatial heterogeneity of global vegetation drought changes may be high in the future. The western United States, South America, central and southern Australia, and southwestern Asia are regions where VHI_opt_ will decreases. On the other hand, central Africa, India, southern China, North America, and high-latitude parts of Asia are areas where vegetation droughts will be alleviated and vegetation health levels will improve. These changes have passed the significance assessment in the MK trend test. In the context of climate change, the changes in vegetation drought in areas where VHI_opt_ has declined are subject to large uncertainties and required more attention.

## Usage Notes

We produced a long-term (1981–2021) high-spatial-resolution (4 km) improved vegetation drought detection dataset and the best contribution parameter dataset that can be used to calculate improved VHIs in different regions. We also evaluated the efficiency of the VHI_opt_ dataset to detect drought and the spatial pattern and changing trends of global vegetation drought to improve our techniques for long-term monitoring of vegetation drought. Based on the best global contribution parameter dataset, VHI_opt_ data in different regions can be calculated for performing drought assessment and studying food production and vegetation carbon sinks.

The data set can help people to carry out drought assessment more conveniently and efficiently, but there are areas where improvements are needed. First, the spatial resolution of the data may have an impacts on findings^57^. Using higher-spatial-resolution remote sensing products and sc-PDSI data will help further improve the detection efficiency of vegetation drought. Second, increased human activities may complicate changes in vegetation aridity^25^. Human interventions may have more complex impacts on vegetation health, requiring further research^58^.

Our research was conducted on a global scale, providing a tool aimed at understanding global and regional vegetation drought characteristics. This dataset greatly improves the ability of VHI to detect vegetation drought, and can help people better understand the impact of temperature on vegetation drought in different regions. This research is also conducive to the effective implementation of vegetation drought resistance ecological engineering, and helps local governments and farmers reduce the losses caused by vegetation drought. The global vegetation drought dataset developed here also has the potential to be used to monitor and assess drought and its impact on the agricultural, forestry, ecological, and environmental sectors.

## Data Availability

All calculations of a global long term (1981–2021), high resolution (4 km) improved vegetation health index (VHI) dataset are based on MATLAB, and relevant step codes can be obtained from Github: https://github.com/BNUJingyuZeng/A-new-global-VHI-dataset-code.git.

## References

[1] Van Loon AF (2015). Hydrological drought explained. Wiley Interdisciplinary Reviews-Water.

[2] Hameed M, Ahmadalipour A, Moradkhani H (2020). Drought and food security in the middle east: An analytical framework. Agricultural and Forest Meteorology.

[3] Kogan F, Guo W, Yang WZ (2019). Drought and food security prediction from NOAA new generation of operational satellites. Geomatics Natural Hazards & Risk.

[4] Dadson, S. J., Lopez, H. P., Peng, J. & Vora, S. in *Water Science, Policy, and Management* 11–28 (2019).

[5] Marvel K (2019). Twentieth-century hydroclimate changes consistent with human influence. Nature.

[6] Bento VA, Russo A, Vieira I, Gouveia CM (2023). Identification of forest vulnerability to droughts in the Iberian Peninsula. Theoretical and Applied Climatology.

[7] Zhang, R. *et al*. The first high spatial resolution multi-scale daily SPI and SPEI raster dataset for drought monitoring and evaluating over China from 1979 to 2018. *Big Earth Data*, 1–26, 10.1080/20964471.2022.2148331 (2023).

[8] AghaKouchak A (2015). Remote sensing of drought: Progress, challenges and opportunities. Reviews of Geophysics.

[9] AghaKouchak A (2015). A multivariate approach for persistence-based drought prediction: Application to the 2010–2011 East Africa drought. Journal of Hydrology.

[10] Wu X (2022). The Effect of Drought on Vegetation Gross Primary Productivity under Different Vegetation Types across China from 2001 to 2020. Remote Sensing.

[11] Wu Z (2020). Recent changes in the drought of China from 1960 to 2014. International Journal of Climatology.

[12] Lesk C, Rowhani P, Ramankutty N (2016). Influence of extreme weather disasters on global crop production. Nature.

[13] Wang Q (2014). Temporal-spatial characteristics of severe drought events and their impact on agriculture on a global scale. Quaternary International.

[14] Zhang Z (2014). Spatial pattern and decadal change of agro-meteorological disasters in the main wheat production area of China during 1991–2009. Journal of Geographical Sciences.

[15] He B, Huang L, Wang Q (2015). Precipitation deficits increase high diurnal temperature range extremes. Scientific Reports.

[16] He B, Wang HL, Wang QF, Di ZH (2015). A quantitative assessment of the relationship between precipitation deficits and air temperature variations. Journal of Geophysical Research: Atmospheres.

[17] Johansson R, Luebehusen E, Morris B, Shannon H, Meyer S (2015). Monitoring the impacts of weather and climate extremes on global agricultural production. Weather and Climate Extremes.

[18] Wang Q (2021). A multi-scale daily SPEI dataset for drought characterization at observation stations over mainland China from 1961 to 2018. Earth Syst. Sci. Data.

[19] Wang Q (2022). An improved daily standardized precipitation index dataset for mainland China from 1961 to 2018. Scientific Data.

[20] Zhang R (2022). Investigating the effect of improved drought events extraction method on spatiotemporal characteristics of drought. Theoretical and Applied Climatology.

[21] Wells N, Goddard S, Hayes MJ (2004). A self-calibrating Palmer Drought Severity Index. Journal of Climate.

[22] Palmer, W. C. in *U.S. Weather Bureau, Res. Pap. No. 45* 58–58 (1965).

[23] Zeng J (2022). Improving the drought monitoring capability of VHI at the global scale via ensemble indices for various vegetation types from 2001 to 2018. Weather and Climate Extremes.

[24] Wang Q (2015). The alleviating trend of drought in the Huang-Huai-Hai Plain of China based on the daily SPEI. International Journal of Climatology.

[25] Zeng J (2020). Drought frequency characteristics of China, 1981–2019, based on the vegetation health index. Climate Research.

[26] Wu Z, Yu L, Zhang X, Du Z, Zhang H (2019). Satellite-based large-scale vegetation dynamics in ecological restoration programmes of Northern China. International Journal of Remote Sensing.

[27] Leng S (2022). Assessing the Impact of Extreme Droughts on Dryland Vegetation by Multi-Satellite Solar-Induced Chlorophyll Fluorescence. Remote Sensing.

[28] Zhang R, Qi J, Leng S, Wang Q (2022). Long-Term Vegetation Phenology Changes and Responses to Preseason Temperature and Precipitation in Northern China. Remote Sensing.

[29] Leng S (2022). Spatiotemporal Variations of Dryland Vegetation Phenology Revealed by Satellite-Observed Fluorescence and Greenness across the North Australian Tropical Transect. Remote Sensing.

[30] Tang J (2020). A modified flexible spatiotemporal data fusion model. Frontiers of Earth Science.

[31] Ezzine H, Bouziane A, Ouazar D (2014). Seasonal comparisons of meteorological and agricultural drought indices in Morocco using open short time-series data. International Journal of Applied Earth Observation and Geoinformation.

[32] Li Y, Strapasson A, Rojas O (2020). Assessment of El Niño and La Niña impacts on China: Enhancing the Early Warning System on Food and Agriculture. Weather and Climate Extremes.

[33] Bento VA, Gouveia CM, DaCamara CC, Trigo IF (2018). A climatological assessment of drought impact on vegetation health index. Agricultural and Forest Meteorology.

[34] Kogan, F. N. in *Natural* Haza*rd*s: Mo*nitoring and Assessment Using Remote Sensing Technique* Vol. 15 *Advances in Space Research-Series* (eds Singh, R. P. & Furrer, R.) 91–100 (1995).

[35] Kogan FN (1997). Global drought watch from space. Bulletin of the American Meteorological Society.

[36] Bhuiyan C, Singh RP, Kogan FN (2006). Monitoring drought dynamics in the Aravalli region (India) using different indices based on ground and remote sensing data. International Journal of Applied Earth Observation and Geoinformation.

[37] Tang J (2020). Self-adapting extraction of cropland phenological transitions of rotation agroecosystems using dynamically fused NDVI images. International Journal of Biometeorology.

[38] Yousef F, Gebremichael M, Ghebremichael L, Perine J (2019). Remote-sensing Based Assessment of Long-term Riparian Vegetation Health in Proximity to Agricultural Lands with Herbicide Use History. Integrated Environmental Assessment and Management.

[39] Bento VA, Gouveia CM, DaCamara CC, Libonati R, Trigo IF (2020). The roles of NDVI and Land Surface Temperature when using the Vegetation Health Index over dry regions. Global and Planetary Change.

[40] Bi, W. *et al*. A global 0.05 degrees dataset for gross primary production of sunlit and shaded vegetation canopies from 1992 to 2020. *Scientific Data* **9**, 10.1038/s41597-022-01309-2 (2022). 10.1038/s41597-022-01309-2 PMC9110750 35577806

[41] Liu XF (2023). Compound droughts slow down the greening of the Earth. Global Change Biology.

[42] Vicente-Serrano SM (2013). Response of vegetation to drought time-scales across global land biomes. Proceedings of the National Academy of Sciences of the United States of America.

[43] Zhu, X. R. *et al*. Soil coarsening alleviates precipitation constraint on vegetation growth in global drylands. *Environmental Research Letters* **17**, 10.1088/1748-9326/ac953f (2022).

[44] Dunn RJH (2022). Global Climate. Bulletin of the American Meteorological Society.

[45] van der Schrier G, Barichivich J, Briffa KR, Jones PD (2013). A scPDSI-based global data set of dry and wet spells for 1901–2009. Journal of Geophysical Research: Atmospheres.

[46] EM-DAT. *CRED/UCLouvain*, <Brussels, Belgium> (www.emdat.be).

[47] Zeng J (2022). figshare.

[48] Karnieli A (2010). Use of NDVI and Land Surface Temperature for Drought Assessment: Merits and Limitations. Journal of Climate.

[49] Monteleone B, Bonaccorso B, Martina M (2020). A joint probabilistic index for objective drought identification: the case study of Haiti. Nat. Hazards Earth Syst. Sci..

[50] Kogan F, Salazar L, Roytman L (2012). Forecasting crop production using satellite-based vegetation health indices in Kansas, USA. International Journal of Remote Sensing.

[51] Zeng J (2021). Ecological sustainability assessment of the carbon footprint in Fujian Province, southeast China. Frontiers of Earth Science.

[52] Wang Q (2021). Pronounced Increases in Future Soil Erosion and Sediment Deposition as Influenced by Freeze–Thaw Cycles in the Upper Mississippi River Basin. Environmental Science & Technology.

[53] Wang Q, Tang J, Zeng J, Leng S, Shui W (2019). Regional detection of multiple change points and workable application for precipitation by maximum likelihood approach. Arabian Journal of Geosciences.

[54] Wang Q (2018). The effects of air temperature and precipitation on the net primary productivity in China during the early 21st century. Frontiers of Earth Science.

[55] Mann HB (1945). Nonparametric Tests Against Trend. Econometrica.

[56] Kendall MG (1990). Rank correlation methods. British Journal of Psychology.

[57] Jay S (2019). Exploiting the centimeter resolution of UAV multispectral imagery to improve remote-sensing estimates of canopy structure and biochemistry in sugar beet crops. Remote Sensing of Environment.

[58] Kou PL (2021). Complex anthropogenic interaction on vegetation greening in the Chinese Loess Plateau. Science of the Total Environment.

